# A retrospective evaluation of point of care ultrasound for acute cholecystitis in a tertiary academic hospital setting

**DOI:** 10.1186/s13089-021-00228-4

**Published:** 2021-06-03

**Authors:** David P. Evans, Jordan Tozer, Lindsay Taylor, Michael J. Vitto, Michael Joyce

**Affiliations:** Department of Emergency Medicine, Virginia Commonwealth School of Medicine, Main Hospital 2nd floor, room 606, Suite 600, 1250 East Marshal St, PO BOX 980401, Richmond, VA 23298-0401 USA

**Keywords:** Biliary ultrasound, Medical education, Cholecystitis

## Abstract

**Background:**

In 2008 the Council of Emergency Medicine Residency Directors delineated consensus recommendations for training in biliary ultrasound for the “detection of biliary pathology”.

**Objectives:**

While studies have looked at the accuracy of emergency provider performed clinical ultrasound (ECUS), we sought to evaluated if ECUS could be diagnostic for acute cholecystitis and thus obviate the need for follow-up imaging.

**Method:**

We reviewed all ECUS performed between 2012 and 2017 that had a matching radiology performed ultrasound (RADUS) and a discharge diagnosis. 332 studies were identified. The sensitivity and specificity of both ECUS and RADUS were compared to the patient’s discharge diagnosis. The agreement between the ECUS and RADUS was assessed using an unweighted Cohen’s Kappa. The time from patient arrival to diagnosis by ECUS and RADUS was also compared.

**Results:**

Using discharge diagnosis as the gold standard ECUS was 67% (56–78%) sensitive, 88% (84–92%) specific, NPV 90% (87–95%), PPV 60% (50–71%), +LR 5.6 (3.9–8.2), −LR 0.37 (0.27–0.52) for acute cholecystitis. RADUS was 76% (66–87%) sensitive, 97% (95–99%) specific, NPV 95% (092–97%), PPV 86% (76–95%), +LR 25.6 (12.8–51.4), and −LR 0.24 (0.15–0.38). ECUS was able to detect gallstones with 93% (89–96%) sensitivity and 94% (90–98%) specificity leading to a NPV 90% (85–95%), PPV of 95% (92–98%), +LR 14.5 (7.7–27.4), −LR 0.08 (0.05–0.13). The unweighted kappa between ECUS and RADUS was 0.57. The median time between obtaining ECUS vs. RADUS diagnosis was 124 min.

**Conclusions:**

ECUS can be beneficial in ruling out acute cholecystitis, but lacks the test characteristics to be diagnostic for acute cholecystitis.

## Introduction

### Background

Cholecystitis afflicts over 20 million people in the United States [1]. Amongst people with biliary disease approximately 1–4% develop biliary colic annually [2–5]. Of these symptomatic patients, 20% will go on to develop a potentially life-threatening complication such as acute cholecystitis [6]. This comes at a direct annual cost to the U.S. health care system of 6.3 billion dollars [7]. Unfortunately, historical and physical exam findings lack the ability to identify acute cholecystitis in the clinical setting [8]. Given this, clinicians in the emergency department (ED) routinely order radiology-performed ultrasound (RADUS) of the gallbladder to “rule out” acute cholecystitis. RADUS is typically performed by an ultrasound technician and interpreted by a radiologist. This approach leads to inherit lag times in radiology-based imaging that prolong ED wait times causing throughput delays and contributing to overcrowding [9, 10]. Due to these issues, emergency physicians have begun to explore the use of clinician-performed ultrasound (ECUS) to evaluate patients for possible biliary disease [11–13]. This led to the 2008 Council of Emergency Medicine Residency Directors consensus recommendations for training in biliary ultrasound for the “detection of biliary pathology” [14]. Following these recommendations it became commonplace at our institution for resident physicians to perform a ECUS of the gallbladder for any patient presenting with right upper quadrant or epigastric pain. It is also common at our institution for an attending physician working in the triage area to order formal right upper quadrant scans through radiology, while the patient is in triage.

### Importance

Following these recommendations, academic emergency departments began to teach clinical ultrasound to all residents, requiring a basic competency prior to graduation. In the decade, since these recommendations were made, few studies have looked at the accuracy of emergency physician performed clinical ultrasound (ECUS) and none have evaluated if ECUS could be diagnostic for biliary pathology and thus obviate the need for follow-up imaging [15, 16].

### Goals of this investigation

The goal of this investigation was to perform a retrospective chart review study evaluating ECUS test characteristics for acute cholecystitis as compared to the patients discharge diagnosis. Secondary objectives were to evaluate how ECUS correlated to RADUS, the time difference to obtaining ECUS vs. RADUS, the use of cholescintigraphy in the ED, and lastly to evaluate how different clinical ultrasound test characteristics for acute cholecystitis performed.

## Materials and methods

### Study design

This was a retrospective review of historical data retrieved from the ultrasound archiving system (Qpath, Telexy, Canada) of a large urban teaching hospital from January 1, 2012 to January 1, 2017. All studies were performed on Sonosite Xport machines (Fujifilm Sonosite, Bethell, WA). The study was approved by our Institutional Review Board.

### Setting and selection of participants

All emergency physician-performed focused gallbladder studies completed during the eligible period that had a matching radiology-interpreted study and formal discharge diagnosis were included. Patients that underwent HIDA scan for inconclusive radiology-performed ultrasound were included in the data. The time from the initial patient encounter by ED physician to diagnosis by ECUS, RADUS and HIDA was recorded and compared.

### Primary data analysis

#### Detecting pathology

The protocol for all ECUS studies of the gallbladder at our institution consisted of a 4 second clip of the gallbladder both in long and short access, as well as image of the common bile duct. The gallbladder wall is to be measured on the short axis, where it abuts the liver anteriorly with a normal measurement of less than 0.34 cm. The common bile duct is verified with color Doppler and its inner diameter measured in the longitudinal axis with a normal measurement of less than 0.7 cm. Abnormal findings would include gallstones, wall thickening, pericystic fluid, sonographic murphy’s sign or enlargement of the common bile duct. The ECUS studies were grouped into the following diagnostic categories: no pathology, cholelithiasis, cholelithiasis with cholecystitis, acalculous cholecystitis, choledocolithiasis, polyps, and other based solely on the ED physician’s final interpretation. The sensitivity, specificity, positive likelihood and negative likelihood ratios for both ECUS and RADUS were compared using the patient’s discharge diagnosis for the same above categories. The discharge diagnosis was obtained from the patient’s discharge summary. All chart reviews were conducted by DE. Data was collected and stored in Excel (Microsoft, Redmond, WA). All statistical testing was performed with Stata (version 12.1; StataCorp, College Station, Tx).

#### Test for agreement

The agreement between the ECUS diagnosis and the RADUS diagnosis was assessed using an unweighted Cohen’s Kappa. An initial test for agreement used all diagnosis categories and a second test for agreement combined the diagnoses of acalculous cholecystitis, choledocolithiasis, and polyps into the other category, as they had low representations in the data. Cohen’s Kappa was calculated again for the agreement between the two new sets of diagnoses.

#### Time analysis

The time between the initial physician encounter to ECUS diagnosis, RADUS diagnosis, and HIDA diagnosis, was calculated for each patient. Eleven patients were omitted from this analysis, because they received a RADUS study prior to being seen by an emergency physician. Eighty-eight additional patients had missing values for their times and were omitted from the analysis. For the patients that received a HIDA scan, the time from initial encounter to the HIDA scan diagnosis was calculated. The median times to ECUS diagnosis, RADUS diagnosis, and HIDA diagnosis were calculated and compared.

## Results

Of the 3121 total ECUS studies of the gallbladder performed during the study period 332 were identified that met inclusion criteria. Of these, 189 patients were diagnosed with cholelithiasis and 47 were diagnosed with cholecystitis by ECUS, while 131 patients were diagnosed with cholelithiasis and 56 were diagnosed with cholecystitis by RADUS.

When compared to the discharge diagnosis of acute cholecystitis, RADUS was more sensitive (76% vs. 67%) and specific (97% vs. 88%), with better predictive value compared to ECUS (Table 1). When looking at the discharge diagnosis of cholelithiasis ECUS was 92.7% sensitive and 93.6% specific compared to RADUS, which was 95.4% sensitive and 84.1% specific (Table 2). The unweighted kappa between ECUS and RADUS was 0.57.

**Table 1:** EUS vs. RADUS for acute cholecystitis

	EUS (955 CI)	RADUS (95% CI)
Sensitivity	67% (56–78%)	76% (66–87%)
Specificity	88% (84–92%)	97% (95–99%)
NPV	90% (87–95%)	95% (92–97%)
PPV	60% (50–71%)	86% (76–95%)
+LR	5.6 (3.9–8.2)	25.6 (12.8–51.4)
−LR	0.37 (0.27–0.52)	0.24 (0.15–0.38)

**Table 2:** EUS ability to detect gallstones

Sensitivity	93% (89–96%)
Specificity	94% (90–98%)
NPV	90% (85–95%)
PPV	95% (92–98%)
+LR	14.5 (7.7–27.4)
−LR	0.08 (0.05–0.13)

Of the 332 subjects, 83 had surgery with available pathology for review. Of the patients who went to the operating room, 53 were diagnosed with cholecystitis, 25 with cholelithiasis, and 5 with gallstone pancreatitis. Of the 53 patients diagnosed with cholecystitis, 30 (56.6%) were diagnosed on ECUS as having cholecystitis and 22 (41.5%) were diagnosed with gallstones without signs of cholecystitis. In comparison, 37 (70%) were diagnosed on RADUS as having cholecystitis and 13 (24%) were diagnosed with gallstones without signs of cholecystitis.

Acalculous cholecystitis was diagnosed by ECUS in 16 patients and by RADUS in 9 patients. Of the 332 patients studied; however, only one received a discharge diagnosis of acalculous cholecystitis.

In our study population, ECUS deemed 103 patients to have no significant gallbladder pathology. Of these, 12 patients were diagnosed with cholelithiasis on RADUS, one of which had a subsequent HIDA scan that showed no pathology. None of the 103 patients with a negative ECUS were diagnosed with acute cholecystitis and none returned to the study institution in the 6 months after their ED visit with a diagnosis of acute cholecystitis.

Therefore, dichotomizing biliary ultrasound into pathology vs. no pathology ECUS was 93.6% (90–97.1%) sensitive and 67.6% (60–75.2%) specific with positive LR of 2.89 (2.27–3.66) and negative likelihood ratio of 0.09 (0.05–0.17). Conversely, RADUS was 96.3% (93.6–98.9) sensitive and 73.9% (66.7–81.1) specific with a positive LR of 3.69 (2.8–4.88) and negative LR of 0.04 (0.02–0.10).

The median time between acquisition of the ECUS and RADUS was 124 min, while the median time between ECUS and HIDA scan was 800 min.

## Discussion

Biliary disease places a significant burden on the United States healthcare system with up to 20% of the more than 20 million with disease developing potentially life-threatening complications. Knowing that medical history and physical exam cannot reliably identify biliary disease, a rapid but accurate screening test is desirable. This is beneficial for multiple reasons, including early identification of complications, as well as ED throughput. Avoiding the need for further imaging could decrease time to disposition, and improve patient outcomes through earlier identification of significant complications and thus earlier intervention. When compared to RADUS, we found that ECUS was slightly less sensitive (67% vs. 76%) and specific (88% vs. 97%) for acute cholecystitis when using discharge diagnosis as the gold standard.

In the 17 patients who received HIDA scan none had a change in their diagnosis. The average delay in diagnosis between HIDA and ECUS was over 13 h. This result is consistent with previous studies showing no benefit to HIDA scan in the ED [17].

After dichotomization of patients into those with pathology and those with no significant sonographic findings by ECUS, the sensitivity of ECUS for determining presence of disease was 93.6% (90–97.1%). None of the 103 patients with negative ECUS had a diagnosis of acute cholecystitis or returned to the study institution within 6 months with acute cholecystitis. This makes ultrasound a valuable screening tool in the hands of the emergency physician, and can allow for earlier discharge of well appearing patients if there is no concern for alternate etiology.

In a patient in which the emergency physician has a high pretest probability for acute cholecystitis, the positive likelihood ratio of 5.6 (3.9–8.2) for ECUS can be considered diagnostic and the emergency physician could possibly forego further imaging prior to surgical consult. Given its lower specificity in detecting pathology in general, it could be argued that ECUS lacks the test characteristics to be diagnostic if the clinical picture is unclear; therefore, a confirmatory radiology study may be considered at the discretion of the emergency physician.

Many EDs around the country are experiencing significant boarding and throughput issues. Previous studies have shown that pelvic ECUS was 66 min faster than RADUS, which lead to a 120-min shorter LOS [18, 19]. At our institution, ECUS lead to a 124-min decrease in time to result compared to RADUS. This finding is in agreement with previous literature that indicates that there could potentially be even more time saved in time to disposition.

Finally, it is prudent to consider the possible financial implication of ECUS in addition to the improved throughput time. Previous work has shown that ECUS programs as a whole, including procedural use, have increased ED revenue by $35,500 with a potential for a $107,700 revenue increase with improved utilization and documentation [20]. This does not take into account the reduction in expenses of formal RADUS. To our knowledge, no study has investigated the potential cost savings of a negative biliary ECUS that does not require a more expensive follow-up RADUS.

This study relied on image acquisition and interpretation by emergency physicians with variable levels of training in performing ECUS. It has been demonstrated in several previous studies that interpretation accuracy improves with increasing number of biliary studies [21], with no improvement over 50 studies [22]. In our study, the attending physician assigned to each case reviewed and attested to the ECUS interpretation prior to it becoming final, per department policy. These physicians were credentialed in ultrasound per the ACEP guidelines, having completed a minimum of 25 prior biliary studies.

Another limitation to consider is the median time to receive the RADUS interpretation may be longer depending on the indication for scan. For example, if a “right upper quadrant” RADUS was ordered this would increase the time the technician would spend obtaining the imaging when compared to a specific “biliary” RADUS. Furthermore, compared to a clinical ultrasound exam fewer views are required to adequately diagnose biliary pathology when compared to a more comprehensive right upper quadrant RADUS.

Like many busy EDs, our emergency department has a physician in triage that may order RADUS studies before the ECUS can be performed. This led to 11 studies being performed by RADUS prior to the treating physician seeing the patient. While these studies were excluded, because no ECUS was performed, accounting for these studies could have shortened the time difference noted between ECUS and RADUS.

The gold standard we used was the diagnosis given to the patient at discharge. While we believe this to be a meaningful patient-oriented outcome, recorded discharge diagnoses are subject to inherit human recording error. To mitigate any discharge diagnosis error that may have occurred, each patient was followed for 6 months by chart review to ensure there was no return to the study institution for repeat assessment. It is conceivable that a follow-up visit occurred elsewhere.

Finally, it should be noted that the retrospective nature of this study opens it to selection bias. The patients who had clear diagnosis made on ECUS could have not received a formal RADUS. Therefore, patients with unclear diagnoses or physically difficult to perform ultrasounds may have been more likely to receive both a ECUS and a RADUS of the gallbladder.

## Conclusions

ECUS is a valuable screening tool in the hands of the emergency physician, which can improve time to diagnosis and potential disposition, in patients in whom biliary colic is suspected. ECUS also has the potential to decreases time to intervention when there is high clinical suspicion of acute cholecystitis. If ECUS is equivocal for a patient in whom the emergency physician has suspicion for biliary pathology, then a confirmatory RADUS should be obtained.

## Data Availability

The data sets used and/or analyzed during the current study are available from the corresponding author on reasonable request.

## Abbreviations

ECUS: Emergency provider performed clinical ultrasound
RADUS: Radiology performed ultrasound
HIDA: Hepatobiliary iminodiacetic acid scan
NPV: Negative predictive value
PPV: Positive predictive value
+LR: Positive likelihood ratio
−LR: Negative likelihood ratio
ACEP: American College of Emergency Medicine
ED: Emergency department
LOS: Length of stay
